# A cross‐sectional study of outcomes for patients undergoing mechanical thrombectomy for pulmonary embolism during 2018–2022: Insights from the PINC AI Healthcare Database

**DOI:** 10.1002/hsr2.2031

**Published:** 2024-04-21

**Authors:** Ripal T. Gandhi, C. Michael Gibson, Wissam A. Jaber

**Affiliations:** ^1^ Miami Cardiac and Vascular Institute Miami Florida USA; ^2^ Division of Cardiovascular Medicine Beth Israel Deaconess Medical Center Boston Massachusetts USA; ^3^ Division of Cardivascular Medicine Emory University School of Medicine Atlanta Georgia USA

**Keywords:** acute pulmonary embolism, mechanical thrombectomy, mortality

## Abstract

**Background and Aims:**

Mechanical thrombectomy (MT) treatments for pulmonary embolism (PE) have yet to be compared directly. We aimed to determine if patient outcomes varied following treatment of PE with different MT devices.

**Methods:**

All PE encounters with an index treatment of MT between January 2018 and March 2022 were analyzed for in‐hospital mortality, discharge to home, and 30‐day readmission outcomes in the PINC AI™ Healthcare Database. MT devices used in each encounter were extracted from hospital charge description free‐text fields using keyword text and fuzzy matching. Unadjusted and adjusted logistic regression was used to model outcomes by device.

**Results:**

A total of 5893 encounters were identified using MT as the sole index PE treatment and 1812 using MT with another treatment. Of these, 41% had insufficient information to identify the devices used (unspecified MT), 33% used the FlowTriever System (large‐bore volume‐controlled aspiration MT), 23% the Indigo System (continuous aspiration MT), and 3% some other MT. Large‐bore volume‐controlled aspiration MT was used with other treatments 13% of the time compared with 23% and 39% for unspecified MT and continuous aspiration MT, respectively. Adjusted logistic regression modeling revealed the odds of in‐hospital mortality were significantly higher for patients treated with unspecified MT ([OR] = 1.42, 95% confidence interval [CI]: [1.10–1.83], *p* = 0.008) or continuous aspiration MT (OR = 1.63, 95% CI: [1.21–2.19], *p* = 0.001) compared with large‐bore volume‐controlled aspiration MT. Discharge to home was significantly lower in these same groups (OR = 0.84, 95% CI: [0.73–0.96], *p* = 0.01, and OR = 0.63, 95% CI: [0.53–0.74], *p* < 0.001, respectively), but readmission risks at 30 days were comparable (OR = 1.08, 95% CI: [0.84–1.38], *p* = 0.56, and OR = 1.20, 95% CI: [0.89–1.62], *p* = 0.24, respectively).

**Conclusion:**

PE outcomes and treatment patterns differ significantly based on the type of MT utilized. Clinical studies directly comparing MT treatments are needed to further understand optimal treatment of PE.

## 1 INTRODUCTION

Pulmonary embolism (PE) is an acute occlusion of pulmonary artery flow, typically resulting from thrombus embolized from a deep vein thrombosis, that occurs in about 115 per 100,000 people in the United States.^1^ The increase in pulmonary artery pressure resulting from PE can cause right ventricle pressure overload and ultimately fatal right heart failure, particularly within the first 72 h of development.^2^ Historically, PE has been treated with anticoagulation (AC), systemic thrombolysis (ST), or surgical embolectomy. However, the development and use of catheter‐directed therapies has increased dramatically in the last two decades. For example, in a national sample of Medicare claims data from 2004 to 2016, Gayou et al. reported a 10‐fold increase in the proportion of PEs treated with catheter‐directed therapies.^3^

Randomized controlled trial data demonstrated that ST reduces PE mortality or hemodynamic decompensation more than AC alone, but does so with an increased risk of major bleeding and hemorrhagic stroke.^4^ Subsequently, two types of catheter directed therapies are being investigated with the goal of lowering the risks associated with PE treatment: catheter‐directed thrombolysis (CDT) and mechanical thrombectomy (MT).^5^, ^6^ While CDT treatments have been shown in a limited randomized study to achieve similar clinical outcomes,^7^ the variety of MT treatments have not yet been compared directly, despite their utilization increasing more than fourfold in the Nationwide Inpatient Sample (NIS) between 2010 and 2018.^8^ Consequently, MT is often assessed as a class, particularly in real‐world data sources where procedure codes do not distinguish between the various MT devices.^9^ However, data from a meta‐analysis suggested significant heterogeneity in mortality, major bleeding, and combined use with thrombolytics following treatment with different types of MT.^10^ Additional evaluation of PE patient outcomes at the device level following MT treatment is warranted to explore these findings.

The PINC AI™ Healthcare Database (PHD) is a national, all‐payer database that contains procedural coding data as well as chargemaster (CDM) data, allowing for identification of specific devices used within a hospital encounter. Using this database, we aimed to determine what, if any, differences existed in patient outcomes following MT treatment of PE with different devices to better understand the optimal treatment of PE using MT.

## 2 METHODS

### 2.1 Data source

The PHD is a national, all‐payer database that contains roughly 25% of all inpatient hospitalizations in the United States.^11^ More than 1300 hospitals/healthcare systems from 45 US states and the District of Columbia have contributed to the database since 2000. The PHD contains data that are deidentified and HIPAA‐compliant in accordance with the HIPAA Privacy Rule, per 45 CFR 164.514(b)(1) through the “Expert Determination” method. As such, this analysis does not meet the definition of human subjects research requiring investigational review board (IRB) review and patient consent.

The PHD contains information about patient characteristics, drugs, devices and other treatments, disease states, costs and resource utilization, and clinical outcomes. Importantly, the PHD also contains the CDM, a comprehensive table of items billable to a hospital patient or health insurance provider. It includes hospital services, medical procedures, equipment fees, supplies, and drugs, documented by service day. The charge description is an optionally completed free‐text field that can be used to extract mentions of specific medical devices and treatments.

Data used in this study included inpatient hospital encounters with a PE diagnosis in any position, where the patient was discharged between January 2018 and March 2022. Data were analyzed at the encounter level and excluded encounters where the patient was less than 18 years old, pregnant, or contained intra‐hospital transfers that did not allow the data to be distinguished at the encounter level. Cost data were adjusted for inflation using healthcare inflation data from the US Labor Department's Bureau of Labor Statistics.^12^ Inflation rates were aggregated quarterly, taking the maximum rate per quarter, and applied to total patient costs from the cohorts.

### 2.2 Device identification and grouping

Two MT devices are currently indicated for use in the treatment of PE in the United States: the Indigo System (Penumbra Inc) and the FlowTriever System (Inari Medical). The Indigo System utilizes pump‐driven continuous aspiration through 6–16 French catheters for thrombus removal (continuous aspiration MT) while the FlowTriever System employs a manually loaded 60 cc vacuum syringe to generate suction through 16–24 French catheters (large‐bore volume‐controlled aspiration MT). Note that the 16‐French Indigo System catheter was released after the data window for this work and so is not represented in this analysis. Identification of the device(s) used in each encounter was extracted from hospital CDM description free‐text fields using a combination of keyword text matching (e.g., “FlowTriever”) and fuzzy matching (e.g., “FlowTr*ver”) for these and other MT device names used in the treatment of PE. In many cases, MT was listed in the International Classification of Diseases, Tenth Revision, Procedure Coding System (ICD‐10‐PCS) codes, but no further information regarding the specific MT device used was available in the CDM. Results for this population are shown under the category “unspecified MT.”

### 2.3 Outcomes, procedures, and adjustment variables

The three clinical outcomes examined in this analysis included in‐hospital all‐cause mortality, discharge to home, and inpatient hospital readmission within 30 days of discharge. Discharge status, including mortality and discharge to home, are included directly in the PHD. Readmission is a derived variable that is only captured if a patient is readmitted to the same hospital or hospital system.

A combination of ICD‐10‐PCS codes and the detailed billing CDM were used to identify PE encounters with interventions by surgery, MT, CDT, or ST (Supporting Information S1: Table S1). The first procedure to occur during an encounter established the index procedure day. To reduce confounding from multiple procedures occurring across a potentially long hospitalization and focus the analysis on procedures most likely to be treating the index PE, only procedures conducted on the index procedure day or within the following 4 days were analyzed. Index day interventions without any other procedure types billed within this window were labeled as standalone. Index day interventions with additional procedure types billed within this window were defined as combination therapy.

International Classification of Diseases, Tenth Revision, Clinical Modification (ICD‐10‐CM) codes were used to examine conditions and diagnoses present during each encounter. ICD‐10‐CM codes used in this analysis were selected based on commonly accepted code sets^13^, ^14^, ^15^ and are shown in Supporting Information S1: Table S1.

### 2.4 Statistical analysis

The goal of this analysis was to compare three clinical outcomes for different types of MT treatments. Descriptive statistics are shown for encounters treated with MT alone (no other treatments within the 4 days following the index procedure), and MT in combination with other treatments occurring on the index procedure day or within the following 4 days. The PHD only contains information on treatment timing by day, limiting more detailed description of the clinical decision pathway.

When MT was the only index treatment, descriptive statistics are shown for large‐bore volume‐controlled aspiration MT, continuous aspiration MT, and unspecified MT. Logistic regression was used to model in‐hospital mortality, discharge to home, and 30‐day readmission. Discharge to home and 30‐day readmission were modeled for patients who did not die in hospital. Unadjusted and adjusted results are shown; the demographics and conditions added to the logistic model are listed in Supporting Information S1: Table S2. All statistical tests were two‐sided with an alpha level of 0.05, conducted in R studio (Version 2023.12.1).

## 3 RESULTS

**Figure 1:**
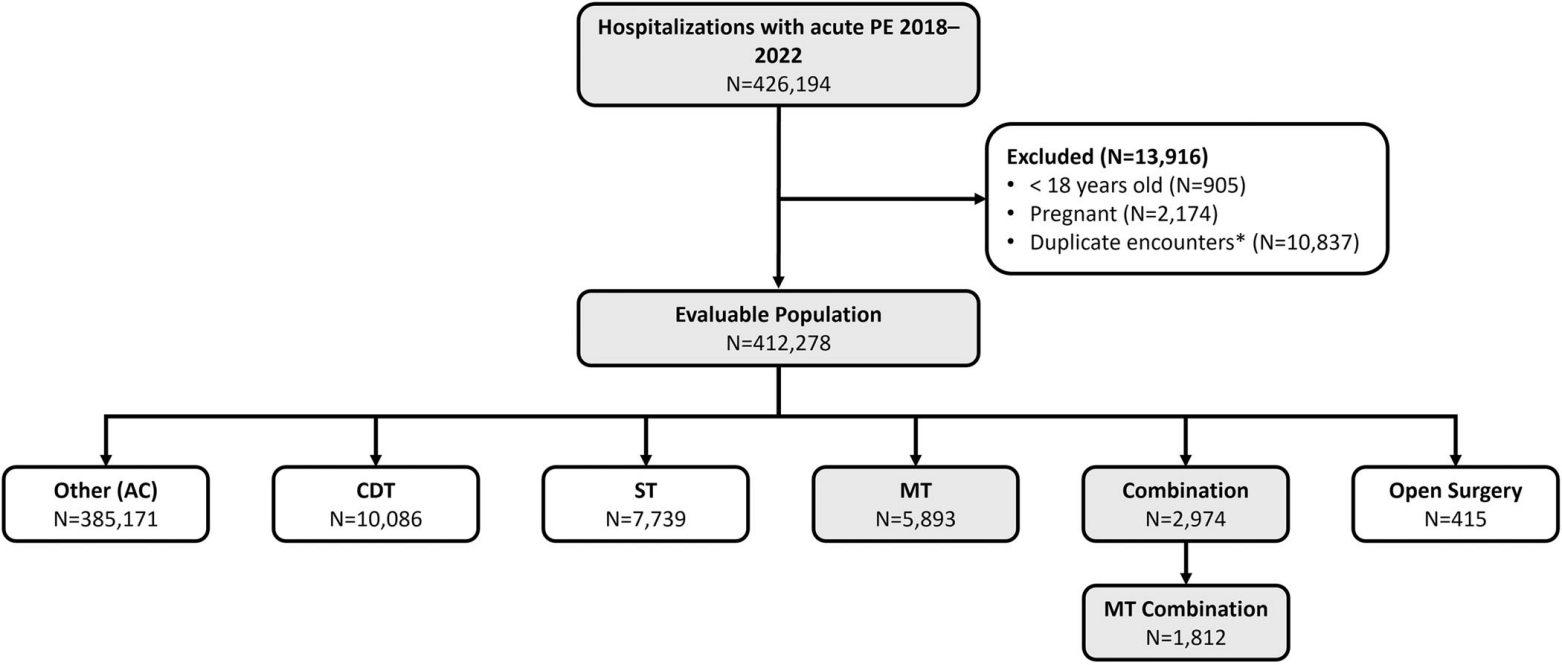
Analysis population. After excluding pregnant patients, those younger than 18 years of age, and *patients with intrahospital transfers leading to duplicate database encounters, all patients hospitalized with acute PE between January 2018 and March 2022 receiving mechanical thrombectomy treatment either alone or in combination with other therapies comprised the analysis population. Patients without treatment codes, “Other,” were presumed to have received AC therapy. AC, anticoagulation; CDT, catheter‐directed thrombolysis; MT, mechanical thrombectomy; PE, pulmonary embolism; ST, systemic thrombolysis.

A total of 426,194 hospitalizations with acute PE were identified in the PHD between January 2018 and March 2022. Of these, 13,916 were excluded based on age (<18 years), pregnancy, or as intra‐hospital transfers leading to duplicate database encounters. From the remaining population, ICD‐10‐PCS codes identified 5893 encounters that used MT as the sole index day PE treatment and an additional 1812 encounters using MT in combination with another treatment (Figure 1).

Table 1 details the summary statistics of patients receiving MT alone as well as those receiving another therapy in combination with MT. Patient demographic characteristics and comorbidities were largely similar regardless of treatment type, though patients receiving MT in combination with non‐CDT therapy had a lower median age (56–58 years vs. 63–64 years), patients receiving MT + CDT were least likely to be COVID‐19 positive (2.3% vs. 5.5%–6.6%), and patients treated with MT combined with a non‐thrombolytic therapy more often presented with sepsis (14% vs. 6.3%–7.7%). Of note, patients receiving MT alone were least likely to be admitted to the ICU after treatment (27% vs. 49%–61%) and demonstrated lower in‐hospital mortality than any other group (6.4% vs. 8.1%–17%).

**Table 1:** Mechanical thrombectomy summary statistics by therapy type.

Characteristics	MT (alone) (*n* = 5893)	MT + CDT (*n* = 931)	MT + ST (*n* = 378)	MT + other (*n* = 503)
Age, years (median [Q1–Q3])	64 (52–73)	63 (51–72)	58 (47–70)	56 (42–68)
Female, *N* (%)	2780 (47)	404 (43)	182 (48)	222 (44)
Ethnicity/Race, *N* (%)				
Black	1169 (20)	182 (20)	74 (20)	98 (19)
Hispanic	297 (5.0)	54 (5.8)	30 (7.9)	45 (8.9)
Other	339 (5.8)	46 (4.9)	30 (7.9)	28 (5.6)
White	4088 (69)	649 (70)	244 (65)	332 (66)
Chronic PE, *N* (%)	125 (2.1)	14 (1.5)	7 (1.9)	13 (2.6)
COVID‐19 positive, *N* (%)	326 (5.5)	21 (2.3)	25 (6.6)	28 (5.6)
Any cancer, *N* (%)	777 (13)	76 (8.2)	39 (10)	54 (11)
Obese, *N* (%)	2281 (39)	400 (43)	149 (39)	224 (45)
Peripheral vascular disease, *N* (%)	465 (7.9)	86 (9.2)	41 (11)	77 (15)
Chronic pulmonary disease, *N* (%)	1101 (19)	194 (21)	56 (15)	103 (20)
Sepsis present on arrival, *N* (%)	409 (6.9)	59 (6.3)	29 (7.7)	71 (14)
Posttreatment LOS, days (median [Q1–Q3])	3 (2–6)	4 (3–7)	4 (2–8)	8 (4–16)
Any Posttreatment ICU stay, *N* (%)	1612 (27)	498 (53)	184 (49)	309 (61)
Posttreatment ICU LOS, among population with any Posttreatment ICU stay, days (median [Q1–Q3])	2 (1–4)	2 (1–4)	2 (1–4)	3 (1–8)
In‐hospital mortality, *N* (%)	376 (6.4)	75 (8.1)	66 (17)	55 (11)
Discharge to home, among population without in‐hospital mortality, *N* (%)	3504 (64)	559 (65)	189 (61)	230 (51)
30‐day readmission, among population without in‐hospital mortality, *N* (%)	367 (6.7)	44 (5.1)	26 (8.3)	42 (9.4)
Total encounter cost, $ (median [Q1–Q3])	26,235 (18,540–39,442)	31,846 (22,506–45,902)	37,103 (25,243–57,135)	49,160 (29,535–90,981)

*Note*: The Hispanic indication is a separate binary variable.

Abbreviations: CDT, catheter‐directed thrombolysis; COVID‐19, coronavirus disease of 2019; ICU, intensive care unit; LOS, hospital length of stay; MT, mechanical thrombectomy; PE, pulmonary embolism; ST, systemic thrombolysis.

**Figure 2:**
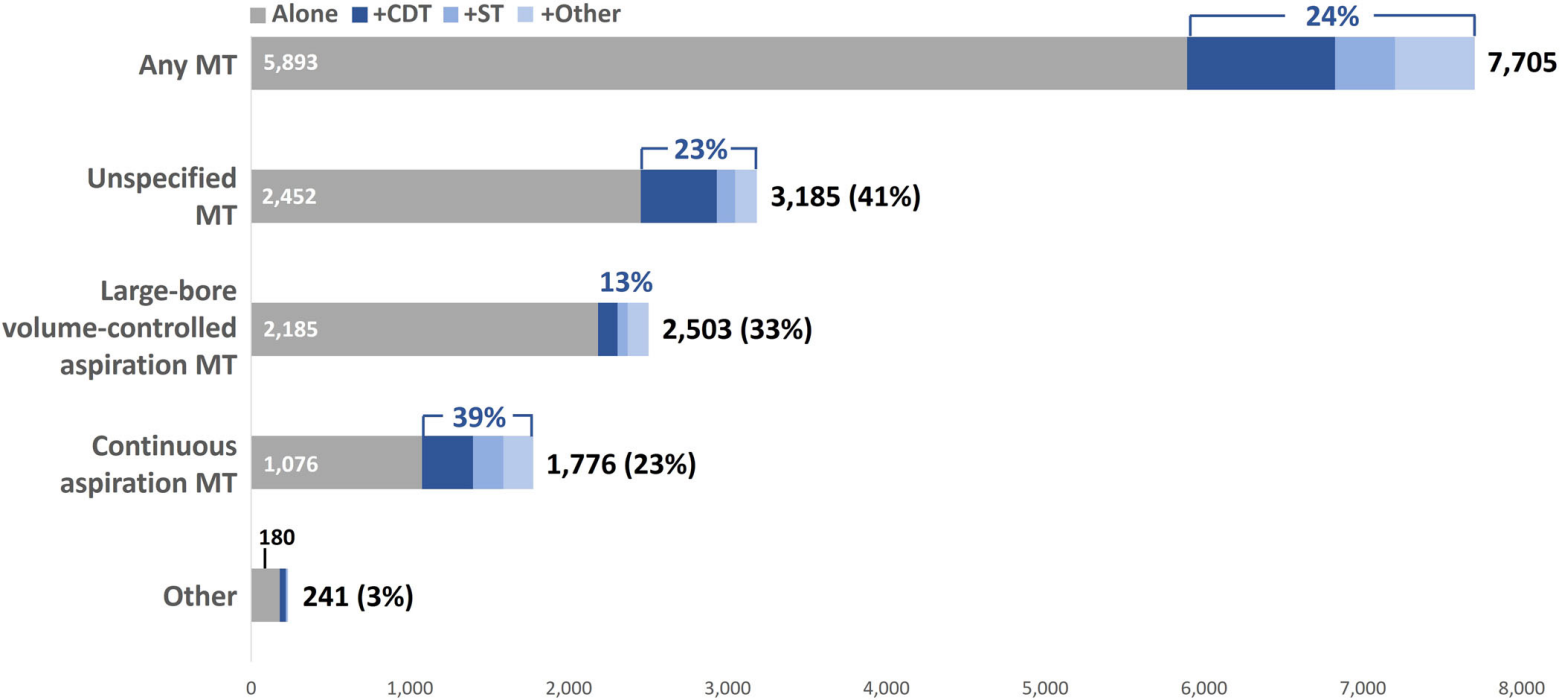
Frequency of standalone MT and use in combination with non‐MT treatments by device. Frequency of standalone treatment (gray) and use in combination with another non‐MT treatment (blue) by MT device. CDT, catheter‐directed thrombolysis; MT, mechanical thrombectomy; ST, systemic thrombolysis.

To explore potential differences in the use of combination therapy between types of MT treatments, keyword text, and fuzzy matching techniques were applied to the hospital CDM field within the PHD to further classify patient treatment by device used. As shown in Figure 2, 41% of MT encounters contained appropriate ICD‐10‐PCS codes but insufficient device‐level hospital charge information for further classification (unspecified MT), 33% of encounters utilized large‐bore volume‐controlled aspiration MT, 23% continuous aspiration MT, and 3.1% some other type of MT system (72% of these being Angiovac, Angiodynamics). Due to their heterogeneity and limited number, encounters in the “other” category were excluded from subsequent analyses. Combination therapy usage varied by MT device. Large‐bore volume‐controlled aspiration MT was used in combination only 13% of the time compared with 23% and 39% for unspecified MT and continuous aspiration MT, respectively. Similarly, thrombolytics were used in conjunction with 29% of continuous aspiration MT encounters, 19% of unspecified MT encounters, and 7.4% of large‐bore volume‐controlled aspiration MT encounters.

Since standalone MT treatment is likely to be associated with the fewest treatment decision confounders, we next examined the summary statistics of patients receiving standalone MT by device to identify any differences in outcomes or patient characteristics (Table 2). Demographic and comorbidity characteristics were mostly comparable in patients across device types except that patients treated with continuous aspiration MT were more frequently Hispanic (7.8% vs. 3.3%–5.2%) or diagnosed with peripheral vascular disease (13% vs. 6.0%–7.5%) and less frequently obese (33% vs. 41%). In‐hospital mortality rates varied by device, ranging from 4.9% for large‐bore volume‐controlled aspiration MT to 8.7% for continuous aspiration MT. Discharge to home among those without in‐hospital mortality was lowest for patients treated with continuous aspiration MT (57% vs. 64%–68%), but 30‐day readmission risks were similar between device treatment groups (6.1%–7.7%).

**Table 2:** Standalone mechanical thrombectomy summary statistics by device.

Characteristics	Large‐bore volume‐controlled aspiration MT (*n* = 2185)	Continuous aspiration MT (*n* = 1076)	Unspecified MT (*n* = 2452)
Age, years (median [Q1–Q3])	64 (52‐72)	63 (51‐73)	64 (53‐73)
Female, *N* (%)	991 (45)	520 (48)	1167 (48)
Ethnicity/Race, *N* (%)			
Black	412 (19)	207 (19)	525 (21)
Hispanic	72 (3.3)	84 (7.8)	128 (5.2)
Other	98 (4.5)	72 (6.7)	153 (6.2)
White	1,603 (73)	713 (66)	1646 (67)
Chronic PE, *N* (%)	50 (2.3)	10 (0.9)	64 (2.6)
COVID‐19 positive, *N* (%)	126 (5.8)	65 (6.0)	133 (5.4)
Any cancer, *N* (%)	279 (13)	174 (16)	301 (12)
Obese, *N* (%)	895 (41)	359 (33)	995 (41)
Peripheral vascular disease, *N* (%)	164 (7.5)	142 (13)	147 (6.0)
Chronic pulmonary disease, *N* (%)	367 (17)	220 (20)	473 (19)
Sepsis present on arrival, *N* (%)	111 (5.1)	95 (8.8)	115 (4.7)
Posttreatment LOS, days (median [Q1–Q3])	3 (2–6)	4 (2–7)	3 (2–6)
Any posttreatment ICU stay, *N* (%)	580 (27)	328 (30)	634 (26)
Posttreatment ICU LOS, among population with any posttreatment ICU stay, days (median [Q1–Q3])	2 (1–3)	2 (1–5)	2 (1–4)
In‐hospital mortality, *N* (%)	106 (4.9)	94 (8.7)	165 (6.7)
Discharge to home, among population without in‐hospital mortality, *N* (%)	1423 (68)	555 (57)	1465 (64)
30‐day readmission, among population without in‐hospital mortality, *N* (%)	126 (6.1)	76 (7.7)	148 (6.5)
Total encounter cost, $ (median [Q1–Q3])	27,255 (20,349–39,861)	27,357 (19,236–41,096)	23,542 (15,926–35,059)

*Note*: The Hispanic indication is a separate binary variable.

Abbreviations: COVID‐19, coronavirus disease of 2019; ICU, intensive care unit; LOS, hospital length of stay; MT, mechanical thrombectomy; PE, pulmonary embolism.

**Figure 3:**
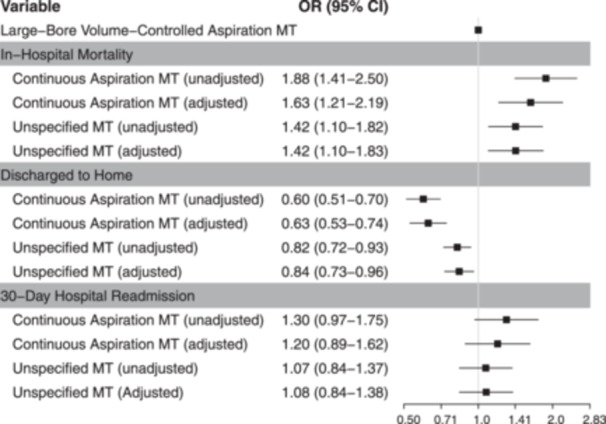
Adjusted and unadjusted logistic regression models for standalone mechanical thrombectomy outcomes by device. Odds ratios for each outcome were calculated using large‐bore volume‐controlled mechanical thrombectomy as the reference group. For adjusted values, multivariable logistic regression analyses were employed to account for age, sex, ethnicity/race, history of chronic pulmonary embolism, positive diagnosis of coronavirus disease of 2019, any cancer, obesity, peripheral vascular disease, chronic pulmonary disease, and sepsis present on arrival. CI, confidence interval; MT, mechanical thrombectomy; OR, odds ratio.

To understand whether patient population differences contributed to the divergences observed for in‐hospital mortality, we compared patient demographic characteristics and comorbidities by both device and in‐hospital mortality outcome (Table 3). As expected, patients who died were more often older (66–69 years vs. 63–64 years), had cancer (22%–28% vs. 11%–16%), or presented with sepsis on arrival to the hospital (12%–20% vs. 3.7%–7.7%). However, no explanatory differences in characteristics were observed between MT devices in patients who died in‐hospital or those who were alive at discharge. To more fully explore the outcome disparities observed between patients receiving treatment with different MT devices, multivariable logistic regression was used to model in‐hospital mortality, discharge to home, and 30‐day readmission adjusting for known confounders (Figure 3). After adjusting for differences in patient characteristics and comorbidities, the odds of in‐hospital mortality remained significant and higher for patients treated with an unspecified MT treatment (odds ratio [OR] = 1.42, 95% confidence interval [CI]: [1.10–1.83], *p* = 0.008) or continuous aspiration MT (OR = 1.63, 95% CI: [1.21–2.19], *p* = 0.001) compared with large‐bore volume‐controlled aspiration MT. In contrast, the odds of being discharged to home were significantly lower in these same groups (OR = 0.84, 95% CI: [0.73–0.96], *p* = 0.01 for unspecified MT and OR = 0.63, 95% CI: [0.53–0.74], *p* < 0.001 for continuous aspiration MT) compared with large‐bore volume‐controlled aspiration MT. After adjustment, 30‐day readmission risks remained comparable between groups (OR = 1.08, 95% CI: [0.84–1.38], *p* = 0.56 for unspecified MT and OR = 1.20, 95% CI: [0.89–1.62], *p* = 0.24 for continuous aspiration MT). Evaluation of admission point of origin and hospital‐level characteristics in each group revealed small differences which, when included in additional modeling, yielded similar results (Supporting Information S1: Table S3).

**Table 3:** Standalone mechanical thrombectomy summary statistics by device and in‐hospital mortality.

	Alive at discharge			Died in hospital		
Characteristics	Large‐bore volume‐controlled aspiration MT (*n* = 2079)	Continuous aspiration MT (*n* = 982)	Unspecified MT (*n* = 2287)	Large‐bore volume‐controlled aspiration MT (*n* = 106)	Continuous aspiration MT (*n* = 94)	Unspecified MT (*n* = 165)
Age, years (median [Q1–Q3])	63 (52–72)	63 (51–72)	64 (53–73)	66 (58–74)	67 (56–75)	69 (58–76)
Female, *N* (%)	937 (45)	478 (49)	1091 (48)	54 (51)	42 (45)	76 (46)
Ethnicity/Race, *N* (%)						
Black	389 (19)	192 (20)	487 (21)	23 (22)	15 (16)	38 (23)
Hispanic	69 (3.3)	74 (7.5)	115 (5.0)	3 (2.8)	10 (11)	13 (7.9)
Other	93 (4.5)	63 (6.4)	143 (6.3)	5 (4.7)	9 (9.6)	10 (6.1)
White	1528 (73)	653 (66)	1542 (67)	75 (71)	60 (64)	104 (63)
Chronic PE, *N* (%)	46 (2.2)	10 (1.0)	63 (2.8)	4 (3.8)	0 (0)	1 (0.6)
COVID‐19 positive, *N* (%)	119 (5.7)	55 (5.6)	122 (5.3)	7 (6.6)	10 (11)	11 (6.7)
Any cancer, *N* (%)	249 (12)	153 (16)	263 (11)	30 (28)	21 (22)	38 (23)
Obese, *N* (%)	861 (41)	329 (34)	944 (41)	34 (32)	30 (32)	51 (31)
Peripheral vascular disease, *N* (%)	157 (7.6)	126 (13)	130 (5.7)	7 (6.6)	16 (17)	17 (10)
Chronic pulmonary disease, *N* (%)	342 (16)	195 (20)	431 (19)	25 (24)	25 (27)	42 (25)
Sepsis present on arrival, *N* (%)	98 (4.7)	76 (7.7)	84 (3.7)	13 (12)	19 (20)	31 (19)

*Note*: The Hispanic indication is a separate binary variable.

Abbreviations: COVID‐19, coronavirus disease of 2019; MT, mechanical thrombectomy; PE, pulmonary embolism.

## 4 DISCUSSION

This analysis compared device‐level differences in outcomes and treatment patterns among hospitalized PE patients treated with MT in a national all‐payer database. The frequency and pattern of combination therapy differed between devices—large‐bore volume‐controlled MT was used 43%–67% less often in combination and 63%–76% less often with thrombolysis than other MT treatments. No differences in 30‐day readmissions were observed based on device‐level treatment, but patients receiving either unspecified MT or continuous aspiration MT had significantly higher adjusted odds ratios (1.42 and 1.63) for dying in‐hospital and significantly lower adjusted odds ratios (0.84 and 0.63) for being discharged to home compared with those treated with large‐bore volume‐controlled MT.

The overall in‐hospital mortality rate of 6.4% observed for MT alone in this study is higher than the 48‐h mortality rates reported in single‐arm evaluations for the principal MT therapies used in this analysis (0%–1.0%)^16^, ^17^, ^18^ and a meta‐analysis of aspiration MT (3.6%).^10^ This is to some degree expected based on the heterogeneous disease severity, comorbidities, and additional procedures or diagnoses unaccounted for in real‐world data compared with clinical trial data. However, the mortality rates for MT alone in the current analysis also appear lower than the 9.1%–13% in‐hospital mortality published in recent real‐world analyses of PE in the NIS and Nationwide Readmissions Database.^8^, ^9^, ^14^, ^19^ This may reflect variation in the populations in each database, the timeframes evaluated, and most likely differences in treatment categorization in each study (e.g., in‐hospital mortality in this analysis was 8%–17% when including patients receiving multiple treatments, including thrombolytics).

However, it is of great interest that, after adjusting for discrepancies in relevant population, admission point of origin, and hospital characteristics and accounting for the significant variation observed in the use of combination therapy, in‐hospital mortality differed significantly between MT device types; patients receiving unspecified MT or continuous aspiration MT had 42% and 63% higher adjusted odds of dying during the index admission compared with patients treated with large‐bore volume‐controlled aspiration MT. The specific causes of the disparities in mortality between MT devices are unclear from this analysis and warrant detailed study. Patient characteristics or other treatment factors not accounted for in our analysis may have influenced the difference in mortality outcomes. While the principal MT systems evaluated herein both use aspiration as their mechanism of action, each device employs different sizes of catheters and types of aspiration (continuous vs. volume‐controlled). Blood loss is connected to each of these parameters and may be one explanation for the differences shown; however, analysis of bleeding events and blood loss in this type of data lacks detail and is fraught with potential errors. Catheter size and aspiration effectiveness can also be associated with differential ability to remove organized and/or large volumes of thrombus.

Rates of patient discharge to home in this analysis are similar to other published studies of MT at a national scale.^8^, ^9^, ^14^, ^19^ However, device‐level analysis enabled by the availability of CDM data identified patients receiving treatment with large‐bore volume‐controlled aspiration MT as 20%–40% more likely to be discharged to home. This may indicate a difference in perceived patient recovery depending on the MT device used for treatment. Additional research is required to explore this and other potential explanations for this difference.

Thirty‐day readmission risks in this analysis did not differ by device but at 5%–9% were substantially lower than the 15% reported across PE treatments in a recent real‐world data analysis of PE.^14^ This is likely due to differential inclusion of surgical interventions and known limitations in the PHD for identifying readmissions outside of the same hospital or hospital system as the index encounter. A more detailed understanding of differences in readmission risks and time to readmission in PE treated with MT remain areas for future research.

Despite sharing many mechanistic similarities, important device usage patterns are apparent from these data. First, large‐bore volume‐controlled aspiration MT is typically used as a standalone therapy. Second, continuous aspiration MT is used in combination with thrombolytics much more often (29% vs. 7.4%–19%) than other MT treatments. This aligns with other published reports of real‐world continuous aspiration MT usage in combination with thrombolytics in about 20% of patients.^20^, ^21^ Whether these patterns indicate a relative lack of standalone effectiveness for continuous aspiration MT or rather reflect historical practice trends is unclear from these data alone. Besides the possibility of a potential class effect difference between large‐bore volume‐controlled aspiration MT and continuous aspiration MT, the patient populations may have fundamental differences that are not accounted for in the current analysis. For example, although the patients had acute PE as a diagnosis, it is possible that some of the MT treatment was used for non‐PE pathology, such as intracardiac or deep vein thrombus, skewing the results.

The findings of this study are strengthened by a large sample size and the use of explicit device name mentions to enable assessment of device‐level outcomes. However, the limitations of this data set and analytic approach provide important context for interpreting these results. First, selection bias and the potential for coding errors are inherent limitations with any retrospective administrative database analysis. We have reduced some of the potential for procedure coding errors by including CDM‐named device data in our analysis. However, at least half of these data contained no explicit device name and may consequently not be representative of use throughout the entire population. Further, limited clinical details (e.g., disease severity, treatment timing, etc.) preclude meaningful analysis of relative disease severity and post‐procedural complications such as bleeding and treatment deterioration. Third, our modeling adjusted for observed differences in our patient populations, however, unknown confounders and disease characteristics or procedures not analyzed in these data may have influenced the differences observed.

## 5 CONCLUSION

PE outcomes and treatment patterns differ significantly based on the type of MT utilized. Rates of in‐hospital mortality and discharge to home were significantly worse in patients treated with continuous aspiration MT versus large‐bore volume‐controlled MT. Continuous aspiration MT was also associated with the highest rates of combination therapy and thrombolytic use. Clinical studies directly comparing MT treatments are needed to further understand optimal treatment of PE using MT.

## AUTHOR CONTRIBUTIONS

**Ripal T. Gandhi**: Conceptualization; methodology; writing—review and editing. **C. Michael Gibson**: Conceptualization; methodology; writing—review and editing. **Wissam A. Jaber**: Conceptualization; methodology; project administration; writing—review and editing. All authors have read and approved the final version of the manuscript.

## CONFLICT OF INTEREST STATEMENT

Ripal T. Gandhi: Consultant for Argon Medical Devices, Becton Dickinson, Boston Scientific, Cordis, Inari Medical, and Medtronic. Speaker for Inari Medical and Penumbra Inc. C. Michael Gibson: Consultant for Inari Medical. Research grant support from Inari Medical, Boston Scientific, and Penumbra. Wissam A. Jaber: Consultant for Inari Medical, Medtronic, and RapidAI. Educational funds from Abbott and Medtronic. Inari Medical provided access to the PINC AI Healthcare Database for this analysis as well as writing and analytical assistance for the initial draft of the manuscript.

## TRANSPARENCY STATEMENT

The lead author Wissam A. Jaber affirms that this manuscript is an honest, accurate, and transparent account of the study being reported; that no important aspects of the study have been omitted; and that any discrepancies from the study as planned (and, if relevant, registered) have been explained.

## Supporting information

Supporting information.

## Data Availability

The PINC AI Healthcare Database is a proprietary database that can be accessed by purchasing an applicable license.
