# Supplementary material for: A cross‐sectional study of outcomes for patients undergoing mechanical thrombectomy for pulmonary embolism during 2018–2022: Insights from the PINC AI Healthcare Database
Source: Health Sci Rep. 2024 Apr 21;7(4):e2031. doi: 10.1002/hsr2.2031 (PMC11033482; doi:10.1002/hsr2.2031)
Supplement: Supplementary file 1 — Supporting information. [file HSR2-7-e2031-s001.docx]

**Supplemental Table S1. ICD Diagnosis Codes**

| Variable | Description | ICD Codes (* denotes wildcard) |
| --- | --- | --- |
| PE | PE ICD diagnosis code (any position) | i26.9*, i26.0* |
| Catheter-Directed Thrombolysis | Fragmentation of: Pulmonary trunk, right pulmonary artery, left pulmonary artery (percutaneous approach, with or without ultrasound) | 02FP3ZZ, 02FQ3ZZ, 02FR3ZZ, 02FR3Z0, 02FP3Z0, 02FQ3Z0 |
| Mechanical Thrombectomy | Extirpation of Matter from: Pulmonary trunk, right pulmonary artery, left pulmonary artery (percutaneous approach); or extirpation of matter from great vessel using computer-aided mechanical aspiration, percutaneous | 02CP3ZZ, 02CQ3ZZ, 02CR3ZZ, X2CY3T7 |
| Open Surgery | Extirpation of Matter from: Pulmonary trunk, right pulmonary artery, left pulmonary artery (open approach) | 02CP0ZZ, 02CQ0ZZ, 02CR0ZZ |
| Systemic Thrombolysis | Introduction of Other Thrombolytic into Central Artery, Percutaneous Approach | 3E06317 |
| Chronic PE | Encounter had chronic PE ICD diagnosis code (any position) | I27.82* |
| COVID-19 | Encounter had COVID-19 ICD diagnosis code (any position) | U07.1* |
| Any Cancer | Encounter had diagnosis code for Lymphoma, any malignancy, metastatic solid tumor, or solid tumor without metastasis (any position) | Lymphoma: C81.*–C85.*, C88.*, C96.*, C90.0, C90.2  Any malignancy: C00.*–C26.*, C30.*–C34.*, C37.*–C41.*, C43.*, C45.*–C58.*, C60.*–C76.*, C81.*–C85.*, C88.*, C90.*–C97.*  Metastatic solid tumor: C77.*–C80.*  Solid tumor without metastasis: C00.*–C26.*, C30.*–C34.*, C37.*–C41.*, C43.*, C45.*–C58.*, C60.*–C76.*, C97.* |
| Obesity | Encounter had diagnosis code for obesity (any position) | E66.* |
| Peripheral vascular disease | Encounter had diagnosis code for PVD (any position) | I70.*, I71.*, I73.1, I73.8, I73.9, I77.1, I79.0, I79.2, K55.1, K55.8, K55.9, Z95.8, Z95.9 |
| Chronic pulmonary disease | Encounter had diagnosis code for CPD (any position) | I27.8, I27.9, J40.*–J47.*, J60.*–J67.*, J68.4, J70.1, J70.3 |
| Sepsis present on arrival | Encounter had diagnosis code for sepsis (any position) noted to be present upon arrival at the hospital | A40%', 'A41%', 'R65.2%', 'T81.12XA%', 'T81.12XD%', 'T81.12XS%', 'R651%' |

COVID-19 = coronavirus disease 2019, ICD = International Statistical Classification of Diseases and Related Health Problems, PE = pulmonary embolism.

**Supplemental Table S2. Logistic Regression Models**

|  | In-Hospital Mortality  (n=5,713) | | | |  | Discharge to Home  (n=5,348) | | | |  | 30-Day Inpatient Readmission  (n=5,348) | | | |
| --- | --- | --- | --- | --- | --- | --- | --- | --- | --- | --- | --- | --- | --- | --- |
| Variable | Unadjusted  OR (95% CI) | p-value | Adjusted  OR (95% CI) | p-value |  | Unadjusted  OR (95% CI) | p-value | Adjusted  OR (95% CI) | p-value |  | Unadjusted  OR (95% CI) | p-value | Adjusted  OR (95% CI) | p-value |
| Large-Bore Volume-Controlled Aspiration MT (reference) |  |  |  |  |  |  |  |  |  |  |  |  |  |  |
| Continuous Aspiration MT | 1.88  (1.41-2.50) | **<.001** | 1.63  (1.21-2.19) | **.001** |  | 0.60  (0.51-0.70) | **<.001** | 0.63  (0.53-0.74) | **<.001** |  | 1.30  (0.97-1.75) | .08 | 1.20  (0.89-1.62) | .24 |
| Unspecified MT | 1.42  (1.10-1.82) | **.007** | 1.42  (1.10-1.83) | **.008** |  | 0.82  (0.72-0.93) | **.002** | 0.84  (0.73-0.96) | **.01** |  | 1.07  (0.84-1.37) | .58 | 1.08  (0.84-1.38) | .56 |
| Age, years |  |  | 1.02  (1.01-1.02) | **<.001** |  |  |  | 0.96  (0.95-0.96) | **<.001** |  |  |  | 1.00  (0.99-1.01) | .96 |
| Female |  |  | 0.98  (0.78-1.22) | .83 |  |  |  | 0.64  (0.57-0.73) | **<.001** |  |  |  | 1.08  (0.84-1.38) | .52 |
| Ethnicity/Race |  |  |  |  |  |  |  |  |  |  |  |  |  |  |
| White (reference) |  |  |  |  |  |  |  |  |  |  |  |  |  |  |
| Black |  |  | 1.22  (0.93-1.61) | .15 |  |  |  | 0.69  (0.60-0.81) | **<.001** |  |  |  | 1.13  (0.86-1.49) | .37 |
| Hispanic |  |  | 1.44  (0.93-2.23) | .10 |  |  |  | 0.72  (0.54-0.95) | **.02** |  |  |  | 1.11  (0.68-1.81) | .69 |
| Other |  |  | 1.16  (0.74-1.81) | .53 |  |  |  | 0.66  (0.51-0.85) | **.002** |  |  |  | 0.58  (0.32-1.05) | .07 |
| Chronic PE |  |  | 0.89  (0.24-1.48) | .26 |  |  |  | 1.04  (0.69-1.56) | .87 |  |  |  | 1.18  (0.61-2.30) | .62 |
| COVID-19 positive |  |  | 1.32  (0.86-2.01) | .20 |  |  |  | 0.83  (0.64-1.08) | .17 |  |  |  | 0.90  (0.54-1.50)) | .69 |
| Any Cancer |  |  | 2.06  (1.59-2.68) | **<.001** |  |  |  | 0.62  (0.52-0.74) | **<.001** |  |  |  | 2.45  (1.88-3.19) | **<.001** |
| Obese |  |  | 0.83  (0.65-1.05) | .12 |  |  |  | 0.99  (0.87-1.12) | .81 |  |  |  | 0.93  (0.74-1.18) | .56 |
| Peripheral vascular disease |  |  | 1.23  (0.86-1.76) | .25 |  |  |  | 0.71  (0.57-0.89) | **.002** |  |  |  | 1.07  (0.72-1.58) | .75 |
| Chronic pulmonary disease |  |  | 1.34  (1.04-1.74) | **.02** |  |  |  | 0.79  (0.68-0.92) | **.003** |  |  |  | 1.61  (1.25-2.08) | **<.001** |
| Sepsis present on arrival |  |  | 3.74  (2.74-5.11) | **<.001** |  |  |  | 0.20  (0.15-0.27) | **<.001** |  |  |  | 1.64  (1.07-2.50) | **.02** |
| Intercept | 0.05  (0.04-0.06) | **<.001** | 0.01  (0.01-0.03) | **<.001** |  | 2.17  (1.98-2.38) | **<.001** | 66.49  (46.90-94.20) | **<.001** |  | 0.07  (0.05-0.08) | **<.001** | 0.05  (0.03-0.09) | **<.001** |

COVID-19 = coronavirus disease of 2019, CI = confidence interval, MT = mechanical thrombectomy, OR = odds ratio, PE = pulmonary embolism. The Hispanic indication is a separate binary variable.

**Supplemental Table S3. Logistic Regression Models Including Hospital-Level Characteristics and Point of Origin**

|  | In-Hospital Mortality  (n=5,713) | | | |  | Discharge to Home  (n=5,348) | | | |  | 30-Day Inpatient Readmission  (n=5,348) | | | |
| --- | --- | --- | --- | --- | --- | --- | --- | --- | --- | --- | --- | --- | --- | --- |
| Variable | Unadjusted  OR (95% CI) | p-value | Adjusted  OR (95% CI) | p-value |  | Unadjusted  OR (95% CI) | p-value | Adjusted  OR (95% CI) | p-value |  | Unadjusted  OR (95% CI) | p-value | Adjusted  OR (95% CI) | p-value |
| Large-Bore Volume-Controlled Aspiration MT (reference) |  |  |  |  |  |  |  |  |  |  |  |  |  |  |
| Continuous Aspiration MT | 1.88  (1.41-2.50) | **<.001** | 1.68  (1.25-2.27) | **.001** |  | 0.60  (0.51-0.70) | **<.001** | 0.61  (0.51-0.72) | **<.001** |  | 1.30  (0.97-1.75) | .08 | 1.13  (0.84-1.54) | .42 |
| Unspecified MT | 1.42  (1.10-1.82) | **.007** | 1.42  (1.10-1.84) | **.008** |  | 0.82  (0.72-0.93) | **.002** | 0.82  (0.72-0.95) | **.006** |  | 1.07  (0.84-1.37) | .58 | 1.04  (0.81-1.34) | .74 |
| Age, years |  |  | 1.02  (1.01-1.03) | **<.001** |  |  |  | 0.96  (0.95-0.96) | **<.001** |  |  |  | 1.00  (0.99-1.01) | .84 |
| Female |  |  | 0.97  (0.78-1.21) | .77 |  |  |  | 0.65  (0.57-0.73) | **<.001** |  |  |  | 1.09  (0.87-1.36) | .47 |
| Ethnicity/Race |  |  |  |  |  |  |  |  |  |  |  |  |  |  |
| White (reference) |  |  |  |  |  |  |  |  |  |  |  |  |  |  |
| Black |  |  | 1.20  (0.91-1.60) | .20 |  |  |  | 0.69  (0.59-0.81) | **<.001** |  |  |  | 1.08  (0.82-1.43) | .57 |
| Hispanic |  |  | 1.37  (0.88-2.13) | .16 |  |  |  | 0.73  (0.55-0.97) | **.04** |  |  |  | 1.06  (0.64-1.74) | .82 |
| Other |  |  | 1.12  (0.72-1.76) | .62 |  |  |  | 0.69  (0.53-0.897) | **.005** |  |  |  | 0.61  (0.34-1.12) | .11 |
| Chronic PE |  |  | 0.59  (0.23-1.47) | .25 |  |  |  | 1.05  (0.69-1.58) | .83 |  |  |  | 1.21  (0.62-2.37) | .58 |
| COVID-19 positive |  |  | 1.32  (0.87-2.03) | .20 |  |  |  | 0.83  (0.63-1.08) | .17 |  |  |  | 0.90  (0.54-1.51) | .70 |
| Any Cancer |  |  | 2.02  (1.55-2.61) | **<.001** |  |  |  | 0.63  (0.53-0.75) | **<.001** |  |  |  | 2.47  (1.90-3.23) | **<.001** |
| Obese |  |  | 0.83  (0.66-1.06) | .13 |  |  |  | 0.98  (0.86-1.12) | .74 |  |  |  | 0.93  (0.74-1.17) | .54 |
| Peripheral vascular disease |  |  | 1.22  (0.86-1.75) | .27 |  |  |  | 0.72  (0.58-0.90) | **.003** |  |  |  | 1.07  (0.72-1.58) | .75 |
| Chronic pulmonary disease |  |  | 1.34  (1.04-1.73) | **.02** |  |  |  | 0.79  (0.68-0.93) | **.003** |  |  |  | 1.60  (1.24-2.07) | **<.001** |
| Sepsis present on arrival |  |  | 3.81  (2.78-5.21) | **<.001** |  |  |  | 0.20  (0.15-0.27) | **<.001** |  |  |  | 1.60  (1.04-2.46) | **.03** |
| Urban or rural hospital |  |  |  |  |  |  |  |  |  |  |  |  |  |  |
| Rural (reference) |  |  |  |  |  |  |  |  |  |  |  |  |  |  |
| Urban |  |  | 1.28  (0.73-2.24) | .39 |  |  |  | 1.07  (0.81-1.41) | .64 |  |  |  | 1.15  (0.69-1.93) | .59 |
| Teaching Hospital |  |  | 1.03  (0.80-1.32) | .82 |  |  |  | 1.00  (0.87-1.15) | >.99 |  |  |  | 1.07  (0.83-1.37) | .61 |
| Hospital Beds |  |  |  |  |  |  |  |  |  |  |  |  |  |  |
| <100 beds (reference) |  |  |  |  |  |  |  |  |  |  |  |  |  |  |
| 100-199 beds |  |  | 1.50  (0.33-6.90) | .60 |  |  |  | 1.08  (0.53-2.20) | .83 |  |  |  | 0.90  (0.25-3.16) | .86 |
| 200-299 beds |  |  | 1.23  (0.28-5.57) | .78 |  |  |  | 1.14  (0.57-2.28) | .71 |  |  |  | 1.04  (0.31-3.56) | .95 |
| 300-399 beds |  |  | 1.55  (0.34-6.97) | .57 |  |  |  | 1.03  (0.51-2.06) | .95 |  |  |  | 0.93  (0.25-3.20) | .91 |
| 400-499 beds |  |  | 1.69  (0.37-7.73) | .50 |  |  |  | 0.81  (0.40-1.65) | .06 |  |  |  | 0.92  (0.26-3.24) | .90 |
| 500+ beds |  |  | 1.93  (0.43-8.65) | .39 |  |  |  | 0.85  (0.42-1.69) | .63 |  |  |  | 0.87  (0.25-2.96) | .08 |
| Point of Origin |  |  |  |  |  |  |  |  |  |  |  |  |  |  |
| Clinic (reference) |  |  |  |  |  |  |  |  |  |  |  |  |  |  |
| Non healthcare facility |  |  | 1.23  (0.72-2.09) | .45 |  |  |  | 0.77  (0.58-1.02) | .06 |  |  |  | 0.75  (0.49-1.14) | .18 |
| Transfer from diff. hospital |  |  | 1.22  (0.69-2.17) | .50 |  |  |  | 0.68  (0.50-0.92) | **.01** |  |  |  | 0.33  (0.19-0.57) | **<.001** |
| Other |  |  | 1.64  (0.78-3.46) | .19 |  |  |  | 0.26  (0.17-0.40) | **<.001** |  |  |  | 0.99  (0.51-1.96) | >.99 |
| Intercept | 0.05  (0.04-0.06) | **<.001** | 0.01  (0.001-0.03) | **<.001** |  | 2.17  (1.98-2.38) | **<.001** | 94.14  (41.24-214.90.04) | **<.001** |  | 0.07  (0.05-0.08) | **<.001** | 0.06  (0.02-0.28) | **<.001** |

COVID-19 = coronavirus disease of 2019, CI = confidence interval, MT = mechanical thrombectomy, OR = odds ratio, PE = pulmonary embolism. The Hispanic indication is a separate binary variable.
